# The Effect of Carotid Chemoreceptor Inhibition on Exercise Tolerance in Chronic Heart Failure

**DOI:** 10.3389/fphys.2020.00195

**Published:** 2020-03-12

**Authors:** Sophie É. Collins, Devin B. Phillips, M. Sean McMurtry, Tracey L. Bryan, D. Ian Paterson, Eric Wong, Justin A. Ezekowitz, Mary A. Forhan, Michael K. Stickland

**Affiliations:** ^1^Division of Pulmonary Medicine, Faculty of Medicine and Dentistry, University of Alberta, Edmonton, AB, Canada; ^2^Faculty of Rehabilitation Medicine, University of Alberta, Edmonton, AB, Canada; ^3^Faculty of Kinesiology, Sport, and Recreation, University of Alberta, Edmonton, AB, Canada; ^4^Division of Cardiology, Faculty of Medicine and Dentistry, University of Alberta, Edmonton, AB, Canada; ^5^G.F. MacDonald Centre for Lung Health, Covenant Health, Edmonton, AB, Canada

**Keywords:** chronic heart failure, exercise tolerance, carotid chemoreceptor, cardiovascular function, dopamine

## Abstract

**Purpose:**

Chronic heart failure (CHF) is characterized by heightened sympathetic nervous activity, carotid chemoreceptor (CC) sensitivity, marked exercise intolerance and an exaggerated ventilatory response to exercise. The purpose of this study was to determine the effect of CC inhibition on exercise cardiovascular and ventilatory function, and exercise tolerance in health and CHF.

**Methods:**

Twelve clinically stable, optimally treated patients with CHF (mean ejection fraction: 43 ± 2.5%) and 12 age- and sex-matched healthy controls were recruited. Participants completed two time-to-symptom-limitation (TLIM) constant load cycling exercise tests at 75% peak power output with either intravenous saline or low-dose dopamine (2 μg⋅kg^–1^⋅min^–1^; order randomized). Ventilation was measured using expired gas data and operating lung volume data were determined during exercise by inspiratory capacity maneuvers. Cardiac output was estimated using impedance cardiography, and vascular conductance was calculated as cardiac output/mean arterial pressure.

**Results:**

There was no change in TLIM in either group with dopamine (CHF: saline 13.1 ± 2.4 vs. dopamine 13.5 ± 1.6 min, *p* = 0.78; Control: saline 10.3 ± 1.2 vs. dopamine 11.5 ± 1.3 min, *p* = 0.16). In CHF patients, dopamine increased cardiac output (*p* = 0.03), vascular conductance (*p* = 0.01) and oxygen delivery (*p* = 0.04) at TLIM, while ventilatory parameters were unaffected (*p* = 0.76). In controls, dopamine improved vascular conductance at TLIM (*p* = 0.03), but no other effects were observed.

**Conclusion:**

Our findings suggest that the CC contributes to cardiovascular regulation during full-body exercise in patients with CHF, however, CC inhibition does not improve exercise tolerance.

## Introduction

Chronic heart failure (CHF) is a condition where heart function is insufficient to meet metabolic demand and is caused by anatomical or physiological abnormalities of the heart (McMurray et al., 2012). Independent of its etiology, CHF has been linked to heightened sympathetic nerve activity (SNA) (Floras, 1993, 2009; Narkiewicz et al., 1999; Andrade et al., 2015). Initially, increased SNA in CHF may be a beneficial adaptation aimed at maintaining cardiac output (Q) and blood pressure. However, chronically increased SNA leads to further heart function deterioration and is directly related to mortality (Cohn et al., 1984). Increased SNA is provoked by changes in autonomic afferent feedback from desensitized baroreceptors and ergoreceptors (Piepoli et al., 1996) as well as increased chemoreceptor activity and sensitivity (Narkiewicz et al., 1999; Sun et al., 1999a, b) which contributes to the vicious cycle worsening cardiac function and CHF (Schultz et al., 2007).

The carotid chemoreceptors (CC) are located within the carotid body at the bifurcation of the common carotid artery and are sensitized by changes in circulating stimuli including O_2_, CO_2_, reactive oxygen species, and pro-inflammatory cytokines (interleukin 6, tumor necrosis factor α). The CC play an important role in ventilatory control and sympathetic vasoconstrictor outflow (Guyenet, 2000; Ding et al., 2011; Porzionato et al., 2013). Giannoni et al. (2009) found that heightened chemosensitivity to both hypoxia (carotid chemoreceptors) and hypercapnia (central chemoreceptors) results in neurohormonal derangement, ventilatory instability, and ventricular arrhythmias. Importantly, hypersensitivity of the CC has been shown to independently predict mortality in patients with CHF (Ponikowski et al., 2001; Jankowska et al., 2007; Giannoni et al., 2009).

A key feature of CHF is markedly reduced exercise capacity (i.e., reduced peak oxygen uptake: VO_2peak_); which is predictive of mortality (Myers et al., 2002; Conraads et al., 2012). The reduced VO_2peak_ in CHF, however, cannot be fully explained by impaired Q, since a peripheral blood flow limitation has also been demonstrated (Clark et al., 1996; Piepoli et al., 1999; Poole et al., 2012). Stickland et al. found that in canines, CC activity is increased during exercise and contributes to the sympathetic restraint of muscle blood flow both in health and experimental CHF (Stickland et al., 2007), however it remains to be determined if the CC is important in cardiovascular regulation and exercise tolerance in patients with CHF.

Dopamine infused at low doses has been shown to suppress the CC (Lahiri et al., 1980; Goldberg, 1989; Stickland et al., 2007). In patients with CHF, CC inhibition with dopamine has been shown to reduce ventilation (van de Borne et al., 1998), and improve cardiovascular function at rest (Edgell et al., 2015). While the CC appears to be activated/sensitized in CHF, and play a role in vascular regulation at rest, it is unclear whether CC inhibition improves cardiovascular function, ventilatory regulation, and exercise tolerance in CHF. Therefore, the purpose of this study was to evaluate the effects of CC inhibition with low-dose dopamine on exercise tolerance, cardiovascular function and ventilation in patients with CHF. We hypothesized that CC inhibition with low-dose dopamine would result in improved exercise tolerance in participants with CHF secondary to improved cardiovascular function and ventilatory regulation.

## Materials and Methods

### Ethical Approval and Participant Description

The study was approved by the University of Alberta Health Research Ethics Board (Biomedical Panel Pro00000526) and is part of a larger research program evaluating the CC in health and disease. Data from nine of the control participants in the current study are included in a manuscript examining the effects of CC inhibition on exercise tolerance in patients with chronic obstructive pulmonary disease (COPD) (Phillips et al., 2019).

Twelve participants with clinically stable CHF, and twelve age- and sex- matched controls were enrolled in the study. All participants provided written, informed consent. Patients with CHF classified as New York Heart Association (NYHA) functional class I – III, receiving optimal pharmacological treatment (ex: ACE-I/ARB, β-blockers, aldosterone antagonists, and diuretics) with no recent cardiac events within the previous 3 months were recruited. Participants receiving opioids, peripheral dopamine receptor blockers, anxiolytics, and antidepressants, were excluded. Participants with severe renal dysfunction, and severe sleep apnea (STOP-Bang questionnaire score >3, and apnea–hypopnea index >30 as evaluated by overnight sleep monitoring with ApneaLink Plus, ResMed Ltd., Bella Vista, Australia) were also excluded.

### Experimental Protocol

A double blind, randomized, placebo-controlled crossover design was used to investigate the effects of CC inhibition with dopamine on exercise tolerance, cardiovascular function and ventilation during whole-body exercise. The protocol, completed over a period of 3 weeks, consisted of four sessions conducted on separate days. The first visit comprised of informed consent, in-depth medical history, a pulmonary function test, and a symptom limited incremental (20 W⋅2 min^–1^) cardiopulmonary exercise test. The second visit involved a basal chemoreflex assessment. The third and fourth visits consisted of two separate constant work-rate exercise tests to symptom limitation (T_LIM_) at 75% of the maximal work rate using either intravenous (IV) low-dose dopamine or placebo saline infusion (order randomized). Prior to each trial, participants were asked to abstain from vigorous exercise, alcohol, and caffeine for 6 h prior to every visit. Patients with CHF were advised to take their CV medications as prescribed on testing days.

### Pulmonary Function Test

A full pulmonary function test was completed in accordance with current guidelines (MacIntyre et al., 2005; Miller et al., 2005; Wanger et al., 2005), wherein standardized spirometry, diffusing capacity, and lung volumes were obtained. The test was completed using the Vmax metabolic system (Encore229 Vmax, SensorMedics, Yorba Linda, CA, United States).

### Cardiopulmonary Incremental Exercise Testing

The incremental exercise tests were preceded by a 3-minute steady state resting period followed by a 1-minute unloaded cycling period. Participants then began cycling exercise using a step-wise protocol, wherein the work rate was increased every 2-minutes by 20 W. Ratings of perceived breathing and leg discomfort (modified Borg scale) (Borg, 1982), and inspiratory capacity (IC) maneuvers (Guenette et al., 2013) were obtained at steady-state rest, during the last 30 s of every 2-minute stage, and at the end of exercise. During every exercise test, all continuous ventilatory and cardiovascular measurements were collected during the first 30 s of every second minute of each stage and linked to the corresponding perceptual ratings and IC maneuvers to avoid contamination of the expired gas data from the IC maneuvers (Jensen et al., 2008).

All exercise tests were completed on an electronically braked cycle ergometer (Ergoselect II 1200; Ergoline, Blitz, Germany), and cardiorespiratory data were recorded using a metabolic measurement system (Encore229 Vmax, SensorMedics, Yorba Linda, CA, United States). Participants were instrumented with finger pulse oximetry (N-595; Nellcor Oximax, Boulder, CO, United States) to estimate arterial O_2_ saturation, and a 12-lead ECG (CardioSoft, GG Medical Systems, Milwaukee, WI, United States) to record heart rate. Arterial blood pressure was obtained through manual auscultation.

### Basal Chemoreception Session

Basal chemoreception sessions were completed with participants laying on a bed in a semi-supine position while single-lead ECG, brachial blood pressure cuff, and ear-lobe pulse oximeter (N-595; Covidien, Mansfield, MA, United States) were attached and continuously monitored and recorded with a data acquisition system (Powerlab 16/30; ADInstruments, NSW, Australia). Data were stored for subsequent analysis using associated software (LabChart 8.0 Pro; ADInstruments). During the tests, participants wore a nose clip and breathed humidified air (HC 150; Fisher and Paykel Healthcare) through a mouthpiece attached to a pneumotachometer (3700 series; Hans Rudolph, Shawnee, KS, United States) and a gas analyzer (CD-3A and S-3A; AEI Technologies, Pittsburgh, PA, United States). The mouthpiece and pneumotachometer were connected to a continuous flow-through system to allow the researcher to switch from the hypoxic or hyperoxic gas blender systems during the chemoreflex tests. Participants completed an initial 10-minute period of quiet, normoxic breathing to ensure a stable baseline prior to initiation of the chemoreflex assessment.

The transient hyperoxic ventilatory response test was used to quantify CC activity, as previously described (Dejours et al., 1958; Phillips et al., 2018). In short, following a period of normoxic breathing, participants breathed hyperoxia (F_i_O_2_: 1.0) for 2 min, and the test was repeated following one minute at normoxia. The greatest 15-second average reduction in minute ventilation from baseline was used to quantify CC activity. To improve sensitivity for comparing between groups, we combined the average change in ventilation from both bouts of hyperoxia. Participants then completed a 10-minute recovery period.

A hyperoxic progressive hypercapnic rebreathe test was completed to estimate central chemosensitivity (Read, 1967). Elevated central chemosensitivity, combined with increased CC sensitivity (hypoxic ventilatory response), has previously been shown to be a prognostic marker in heart failure (Giannoni et al., 2009). Briefly, a four liter rebreathe bag filled with a hyperoxic gas mixture (FiO_2_ = 0.50, FiCO_2_ = 0.07) was attached to the system. First, inspired PO_2_ was raised to ∼350 mmHg (F_i_O_2_ = 0.5) for 5 min, and during end-expiration, the valve was turned over to the rebreathe bag. Participants continued to rebreathe from the bag until an end-tidal partial pressure of CO_2_ (P_ET_CO_2_) of 55 mmHg was reached or until the participant requested to terminate the test. Central chemosensitivity was subsequently evaluated as the slope relating ventilation to P_ET_CO_2_ calculated using linear regression analysis (Read, 1967).

Following another 10-minute recovery period, the transient hypoxic ventilatory response test was administered to evaluate CC sensitivity (Edelman et al., 1973). During expiration, the researcher turned the gas blender from normoxic to pure nitrogen gas (FiO_2_ = 0.0). Participants inhaled 2–8 breaths of nitrogen gas, followed by a 2–5 minute recovery period. Each number of transient breaths was repeated a minimum of two times to obtain a range of oxygen saturations (SpO_2_: 75–100%). The average of the two largest consecutive breaths yielding the highest ventilation following the hypoxic stimulus was used to calculate the change in ventilation from the one minute baseline immediately preceding the stimulus (Ponikowski et al., 2001). The hypoxic ventilatory response was evaluated as the slope relating the change in ventilation to the change in SpO_2_ (Chua and Coats, 1995; Chua et al., 1996a; Ponikowski et al., 2001).

### Constant Work-Rate Exercise Trials

Baseline physiological measurements were obtained for 3 min in the upright seated position prior to the start of the constant work-rate cycling exercise tests. This was followed by a 1-minute period of unloaded pedaling, and then a rapid increase in workload to 75% of peak work-rate. Exercise endurance time was recorded from the onset of constant load to the point of symptom limitation, or when participants were no longer able to maintain a cadence at or above 50 rpm. Measurement procedures for the constant work rate tests were identical to those of the incremental exercise tests, with the addition of the following procedures. Participants were instrumented with impedance cardiography (Physioflow^®^ PF-05, Manatec Biomedical, France) to estimate Q non-invasively (Bernstein, 1986). The changes in impedance cardiography derived Q have been found to accurately estimate the changes that occur during exercise in patients with CHF (Romano et al., 1996; Crisafulli et al., 2007) and impedance cardiography has been validated against the direct Fick method (Northridge et al., 1990; Belardinelli et al., 1996). Hemoglobin concentration ([Hb]) was measured at the beginning of each experimental session (HemoCue 201 + ; HemoCue AB, Angelholm, Sweden) following IV catheter insertion and immediately after the termination of the constant work-rate exercise test, during active recovery. Oxygen delivery was estimated using Q, SpO_2_ and [Hb] data. Baseline [Hb] measurements were used to calculate $\dot{D}\,O_{2}$ during seated baseline, and the [Hb] measurements obtained during active recovery were used to calculate $\dot{D}\,O_{2}$ at TLIM. Vascular conductance was calculated as Q/mean arterial pressure (MAP). Tissue oxygenation was estimated using near infrared spectroscopy (NIRS; Oxymon MK III, Artinis Medical Systems, Netherlands), which has been previously shown to be an accurate estimation of tissue oxygenation during exercise (Wilson et al., 1989; Boushel et al., 1998; Grassi et al., 1999). Consistent with previous work, the optodes were secured on the left thigh at the vastus lateralis using double-sided tape, ensuring that the optodes were separated by approximately 30 mm allowing for a depth of penetration of 15 mm (Homma et al., 1996). The intensities of incident and transmitted light were recorded continuously and used to estimate the changes in tissue oxygenation from resting baseline. Extreme care was taken to ensure consistent optode placement between trials in an attempt to standardize tissue sampled so as to minimize measurement variability.

### Dopamine/Saline Intervention

Prior to the experimental trials, participants were instrumented with an IV catheter in the left antecubital vein to allow for the continuous infusion of either low-dose dopamine hydrochloride (2 μg⋅kg^–1^⋅min^–1^; Hospira, Lake Forest, IL, United States) or isotonic saline solution (order randomized) administered by a constant-infusion pump (Alaris, San Diego, CA, United States). Both the study participant and the lead researcher were blinded to the experimental condition (saline or dopamine). Only the nurse, supervising physician and research coordinator were aware of the condition.

Low-dose dopamine (i.e., 2 μg⋅kg^–1^⋅min^–1^) was selected because it has previously been shown to effectively inhibit the carotid chemoreceptors in humans (Lahiri et al., 1980; Stickland et al., 2011; Edgell et al., 2015). Dopamine does not interact with the central chemoreceptors as it does not cross the blood brain barrier (Zlokovic, 2008).

### Statistical Analysis

Data are presented as mean ± standard error of measurement (SEM) unless otherwise indicated. For all inferential analyses, the probability of a Type I error was set at 0.05. A three-way, repeated measure analysis of variance (ANOVA) was used to evaluate the effect of: saline versus dopamine (factor A) during exercise on dependent variables (repeated factor) in CHF and controls (fixed factor). Two-way repeated-measures ANOVA was used to evaluate the condition by time interactions in each group. If main effects or interaction effects were found, Tukey pairwise comparisons were completed. Unpaired *T*-tests were used to evaluate the cardiopulmonary responses to incremental exercise, the pulmonary function tests, as well as the ventilatory responses to central and carotid chemoreceptor stimuli between groups. Statistical analyses were completed using Sigmaplot 13.0 (Systat Software, San Jose, CA, United States).

## Results

### Participants

See Table 1, for participant demographics. Patients with CHF and controls were matched for age, sex, weight, and height. Mean ejection fraction (EF) at initial CHF diagnosis was 27.0 ± 3.3% (*n* = 9), while EF at study enrolment was 43.0 ± 2.5% (*n* = 12; with a mean improvement of 15 ± 4.1% in EF since initial diagnosis). Ten study participants had heart failure with reduced ejection fraction (HFrEF), and two participants had heart failure with preserved ejection fraction (HFpEF).

**TABLE 1:** Participant characteristics.

	**Controls**	**CHF**	***P*-value**
Participants	12	12	
Male/Female	8/4	8/4	
Age (years)	58.2 ± 3.8	53.6 ± 3.7	0.13
Height (cm)	168.8 ± 2.0	170.4 ± 2.5	0.48
Mass (kg)	75.1 ± 2.3	85.9 ± 4.2	0.049
BMI (kg⋅m^–2^)	26.5 ± 0.96	29.5 ± 1.0	0.08
Smoking history (pack years)	4.9 ± 2.9	3.7 ± 1.4	0.70
**NYHA Functional Class (*n*)**			
I		5	
II		6	
III		1	
Ejection Fraction (%)		43.0 ± 2.5	
LV mass (g⋅m^–2^)		103.3 ± 6.0	
Diabetes Mellitus	0	3	
Hypertension (SBP > 140)	0	2	
**Medication Use (n)**			
β-blockers	1	10	
ACE-I - ARB	0	12	
Aldosterone antagonists	0	10	
Diuretics	0	6	
Statins	0	5	
**Pulmonary Function**			
FEV_1_ (L)	3.4 ± 0.2	3.0 ± 0.3	0.26
FEV_1_ (% pred)	111.4 ± 3.2	91.1 ± 3.8	<0.001
FVC (L)	4.5 ± 0.3	4.1 ± 0.3	0.41
FVC (% pred)	110.8 ± 3.1	96.8 ± 3.8	0.01
FEV_1_/FVC (%)	75.3 ± 1.6	72.3 ± 2.3	0.30
FEV_1_/FVC (% pred)	99.3 ± 1.7	93.7 ± 2.7	0.09
TLC (L)	6.3 ± 0.3	5.7 ± 0.4	0.22
TLC (% pred)	101.3 ± 2.9	90.6 ± 3.8	0.04
RV (L)	1.8 ± 0.1	1.7 ± 0.1	0.64
FRC (L)	3.5 ± 0.2	2.9 ± 0.3	0.11
IC (% pred)	121.9 ± 6.6	105.3 ± 5.4	0.06
DLCO (% pred)	92.5 ± 5.6	77.6 ± 3.4	0.03

*Data are presented as n or mean ± SEM. BMI, body mass index; NYHA, New York Heart Association; LV, left ventricle; ACE-I, angiotensin-converting-enzyme inhibitor; ARB, angiotensin receptor blocker; FEV_1_, forced expiratory volume in 1 s; FVC, forced vital capacity; TLC, total lung capacity; RV, residual volume; FRC, functional residual capacity; IC, inspiratory capacity; DL_CO_, diffusing capacity of the lung for carbon monoxide.*

### Lung Function and Cardiopulmonary Exercise Test

Pulmonary function and cardiopulmonary exercise test results are displayed in Tables 1, 2. Total lung capacity (TLC), forced expired volume in one second (FEV_1_), forced vital capacity (FVC) and diffusing capacity (DL_CO_) were lower in CHF, while no between-group difference in FEV_1_/FVC ratio was observed.

**TABLE 2:** Peak cardiopulmonary exercise responses.

	**Control**	**CHF**	***P*-value**
Work rate (W)	198 ± 22	127 ± 15	0.01
Work rate (% pred)	134 ± 10	79 ± 6	<0.001
$\dot{V}$O_2_ (mL⋅kg^–1^⋅min^–1^)	38.1 ± 3.5	25.0 ± 2.3	0.01
$\dot{V}$O_2_ (L⋅min^–1^)	2.87 ± 0.29	2.12 ± 0.19	0.05
$\dot{V}$CO_2_ (L⋅min^–1^)	3.15 ± 0.31	2.28 ± 0.20	0.03
RQ	1.10 ± 0.02	1.08 ± 0.01	0.32
$\dot{V}$_E_ (L⋅min^–1^)	106 ± 12	69 ± 5	0.01
P_ET_CO_2_ (mmHg)	32.3 ± 0.6	35.7 ± 1.1	0.02
$\dot{V}$_E_/$\dot{V}$CO_2_	32.8 ± 0.8	31.7 ± 1.2	0.46
Nadir $\dot{V}$_E_/$\dot{V}$CO_2_	28.2 ± 0.9	29.0 ± 1.2	0.40
$\dot{V}$_E_/$\dot{V}$CO_2_ slope	28.3 ± 0.7	27.5 ± 1.4	0.61
f_*B*_ (breaths⋅min^–1^)	41.8 ± 3.1	34.3 ± 2.7	0.14
IC (L)	3.04 ± 0.22	2.88 ± 0.20	0.59
IC% TLC	48.7 ± 2.2	50.2 ± 2.8	0.68
Delta IC	0.69 ± 0.42	0.16 ± 0.14	0.24
HR (beats⋅min^–1^)	159.8 ± 5.4	108.6 ± 9.6	<0.001
SpO_2_ (%)	93.0 ± 1.3	97.0 ± 0.3	0.01
Dyspnea (Borg)	6.8 ± 0.6	7.1 ± 0.4	0.69
Leg discomfort (Borg)	6.7 ± 0.4	8.1 ± 0.3	0.02

*Data are presented as mean ± SEM. HEWR, highest equivalent work rate; $\dot{V}$O_2_, oxygen uptake; $\dot{V}$CO_2_, carbon dioxide production; RQ, respiratory quotient; $\dot{V}$_E_, minute ventilation; P_ET_CO_2_, partial pressure of carbon dioxide; *V̇_E_*/$\dot{V}$CO_2_, ventilatory efficiency; f_*B*_, breathing frequency; IC, inspiratory capacity; IRV, inspiratory reserve volume; HR, heart rate; SpO_2_, oxygen saturation measured by pulse oximeter.*

As compared to controls, patients with CHF had a significantly lower relative VO_2peak_, peak minute ventilation ($\dot{V}$_E_), and heart rate. At peak exercise, patients with CHF had higher arterial oxygen saturation, and perceived leg discomfort as compared to controls. There was no between group difference in the ventilatory response to exercise (i.e., $\dot{V}$_E_/*V̇CO*_2_), IC or dyspnea.

### Central and Carotid Chemoreception

There was no statistically significant difference in resting baseline $\dot{V}$_E_ between groups (*p* = 0.31). The change in $\dot{V}$_E_ in response to transient hyperoxia was not significantly different between groups (CHF: 1.29 ± 0.33 vs. controls: 0.85 ± 0.26 L⋅min^–1^, *p* = 0.31). There was no difference in central chemoreflex responses to the progressive hypercapnic rebreathe test between groups (CHF: 1.59 ± 0.37 vs. controls: 1.66 ± 0.37 L⋅min^–1^⋅mmHg^–1^, *p* = 0.88). Patients with CHF had significantly higher ventilatory responses to the transient hypoxia test (CHF: 0.81 ± 0.17 vs. Control: 0.39 ± 0.05 L⋅min^–1^, *p* = 0.04). These data suggest that the patients with CHF had greater carotid chemosensitivity than controls, but no differences in central chemosensitivity.

### The Effect of Low-Dose Dopamine on Exercise Endurance Time

Experimental trial results are displayed in Tables 3, 4. Dopamine did not have an effect on exercise endurance time in either CHF patients (saline: 13.1 ± 2.4 min vs. dopamine: 13.5 ± 1.6 min; *p* = 0.78) or controls (saline: 10.3 ± 1.2 min vs. dopamine: 11.5 ± 1.3 min; *p* = 0.25). A three-way analysis of variance revealed that there was no significant interaction effect between group (CHF vs. control) and condition (saline vs. dopamine) for exercise endurance time (*p* = 0.653).

**TABLE 3:** Effects of dopamine on physiological and perceptual responses during constant load exercise at 75% peak work-rate in patients with chronic heart failure and healthy controls at 4-minute isotime.

	**Control**			**CHF**		
**Condition**	**Saline**	**Dopamine**	***P*-value**	**Saline**	**Dopamine**	***P*-value**
Power output (W)	149 ± 16	149 ± 16		95.3 ± 11	95.3 ± 11	
**Metabolic**						
$\dot{V}$O_2_ (L⋅min^–1^)	2.39 ± 0.27	2.36 ± 0.27	0.45	1.59 ± 0.13	1.63 ± 0.13	0.23
*V̇C*O_2_ (L⋅min^–1^)	2.56 ± 0.28	2.54 ± 0.26	0.81	1.75 ± 0.14	1.78 ± 0.14	0.41
**Ventilatory/gas exchange**						
$\dot{V}$_E_ (L⋅min^–1^)	76.7 ± 8.1	72.9 ± 6.5	0.15	50.0 ± 3.1	48.4 ± 3.4	0.33
$\dot{V}$_E_/$\dot{V}$CO_2_	30.2 ± 0.7	29.2 ± 0.7	0.50	29.2 ± 0.9	27.6 ± 1.0	0.24
f_*B*_ (breaths⋅min^–1^)	29.5 ± 2.3	29.3 ± 1.5	0.88	28.4 ± 1.4	26.5 ± 1.8	0.16
V_*T*_ (L)	2.59 ± 0.18	2.50 ± 0.18	0.34	1.84 ± 0.15	1.94 ± 0.17	0.21
IC (L)	3.21 ± 0.22	3.22 ± 0.19	0.14	2.94 ± 0.21	3.00 ± 0.17	0.37
IRV,%TLC	9.91 ± 1.6	12.0 ± 1.9	0.21	19.8 ± 1.9	19.3 ± 1.6	0.78
P_ET_CO_2_ (mmHg)	35.2 ± 0.8	37.5 ± 0.7	0.02	37.7 ± 1.4	40.1 ± 1.3	<0.001
SpO_2_ (%)	97.6 ± 0.3	96.6 ± 0.5	0.01	96.2 ± 1.2	96.0 ± 0.7	0.80
**Cardiovascular**						
Q (L⋅min^–1^)	13.5 ± 0.8	13.6 ± 1.0	0.79	8.1 ± 0.6	8.4 ± 0.6	0.51
SV (mL)	91.7 ± 4.4	93.6 ± 6.9	0.71	75.9 ± 5.3	78.3 ± 5.1	0.20
HR (beats⋅min^–1^)	146.6 ± 4.2	144.6 ± 4.7	1.00	106.1 ± 4.0	108.0 ± 5.0	0.61
Q/MAP (L⋅min^–1^⋅mmHg^–1^)	113 ± 6.8	120 ± 9.1	0.30	92.9 ± 7.6	99.8 ± 6.8	0.24
MAP (mmHg)	119 ± 3.4	114 ± 3.5	0.21	87.7 ± 3.0	84.6 ± 3.2	0.15
**Perceptual**						
Dyspnea (Borg)	3.8 ± 0.6	3.5 ± 0.5	0.23	3.3 ± 0.5	2.8 ± 0.3	0.23
Leg discomfort (Borg)	4.7 ± 0.6	4.3 ± 0.5	0.56	4.3 ± 0.6	4.0 ± 0.5	0.41

*Data are presented as mean SEM. V̇O_2_, oxygen consumption; V̇CO_2_, carbon dioxide production; V̇E, minute ventilation; V̇E/V̇CO_2_, ventilatory efficiency; fB, breathing frequency; VT, tidal volume; EELV, end-expiratory lung volume; IRV, inspiratory reserve volume; P_*ET*_CO_2_, end-tidal partial pressure of carbon dioxide; SpO_2_, oxygen saturation measured by pulse oximeter; Q, cardiac output; SV, stroke volume; HR, heart rate; Q/MAP, conductance; MAP, mean arterial pressure.*

**TABLE 4:** Effects of dopamine on physiological and perceptual responses during constant load exercise at 75% max workload in patients with chronic heart failure and healthy controls at time of symptom limitation.

	**Control**			**CHF**		
**Condition**	**Saline**	**Dopamine**	***P*-value**	**Saline**	**Dopamine**	***P*-value**
Time (mins)	10.3 ± 1.2	11.5 ± 1.3	0.16	13.1 ± 2.4	13.5 ± 1.6	0.78
Power output (W)	149 ± 16	149 ± 16		95 ± 11	95 ± 11	
**Metabolic**						
$\dot{V}$O_2_ (L⋅min^–1^)	2.69 ± 0.31	2.63 ± 0.32	0.18	1.86 ± 0.16	1.89 ± 0.15	0.33
*V̇C*O_2_ (L⋅min^–1^)	2.67 ± 0.30	2.66 ± 0.30	0.27	1.93 ± 0.16	1.95 ± 0.16	0.57
**Ventilatory/gas exchange**						
$\dot{V}$_E_ (L⋅min^–1^)	93.8 ± 10.5	92.3 ± 10.0	0.55	62.5 ± 5.1	61.2 ± 5.0	0.42
$\dot{V}$_E_/$\dot{V}$CO_2_	35.5 ± 1.2	35.2 ± 1.0	0.84	32.6 ± 1.2	31.5 ± 1.2	0.43
f_*B*_ (breaths⋅min^–1^)	39.6 ± 2.2	39.3 ± 2.0	0.26	35.1 ± 1.6	34.5 ± 2.5	0.66
V_*T*_ (L)	2.45 ± 0.15	2.33 ± 0.16	0.18	1.84 ± 0.16	1.86 ± 0.17	0.84
IC	3.25 ± 0.22	3.24 ± 0.19	0.90	2.97 ± 0.20	3.02 ± 0.19	0.35
IRV,%TLC	12.6 ± 1.3	14.7 ± 1.8	0.22	20.2 ± 2.9	21.1 ± 1.9	0.60
P_ET_CO_2_ (mmHg)	30.9 ± 1.1	31.0 ± 0.9	0.56	33.6 ± 1.2	34.4 ± 1.1	0.26
SpO_2_ (%)	94.8 ± 0.7	94.8 ± 0.5	1.00	96.5 ± 1.1	95.3 ± 0.9	0.09
**Cardiovascular**						
Q (L⋅min^–1^)	14.7 ± 0.69	15.7 ± 1.1	0.14	8.75 ± 0.82	9.77 ± 0.76	0.03
SV (mL)	92.4 ± 3.8	96.3 ± 7.5	0.23	72.2 ± 6.5	81.1 ± 4.7	0.05
HR (beats⋅min^–1^)	156.1 ± 4.5	158.3 ± 6.0	0.86	117.4 ± 4.0	121.8 ± 5.8	0.24
DO_2_ (L⋅min^–1^)	3.00 ± 0.22	3.34 ± 0.28	0.03	1.74 ± 0.16	1.94 ± 0.17	0.04
Q/MAP (L⋅min^–1^⋅mmHg^–1^)	124.4 ± 6.1	137.7 ± 9.9	0.03	95.2 ± 6.8	110.4 ± 7.4	0.01
MAP (mmHg)	118.6 ± 3.4	114.0 ± 2.4	0.23	91.0 ± 3.8	88.9 ± 3.8	0.33
Hb (g⋅dL^–1^)	15.4 ± 0.6	16.1 ± 0.4	0.07	14.8 ± 0.5	14.8 ± 0.5	0.93
**Perceptual**						
Dyspnea (Borg)	7.8 ± 0.5	7.8 ± 0.6	1.00	6.7 ± 0.5	6.8 ± 0.5	0.69
Leg discomfort (Borg)	8.4 ± 0.6	8.7 ± 0.5	0.40	8.2 ± 0.4	8.8 ± 0.3	0.15
Reason for termination						
Legs	5	5		7	9	
Dyspnea	2	2		0	0	
Both	5	5		5	3	

*Data are presented as mean SEM. V̇O_2_, oxygen consumption; V̇CO_2_, carbon dioxide production; V̇_*E*_, minute ventilation; V̇_*E*_/V̇CO_2_, ventilatory efficiency; fB, breathing frequency; VT, tidal volume; EELV, end-expiratory lung volume; IRV, inspiratory reserve volume; PETCO_2_, end-tidal partial pressure of carbon dioxide; SpO_2_, oxygen saturation measured by pulse oximeter; Q, cardiac output; SV, stroke volume; HR, heart rate; DO_2_, oxygen delivery; Q/MAP, conductance; MAP, mean arterial pressure; Hb, hemoglobin concentration.*

### The Effects of Low-Dose Dopamine on the Ventilatory Response to Exercise

**FIGURE 1:**
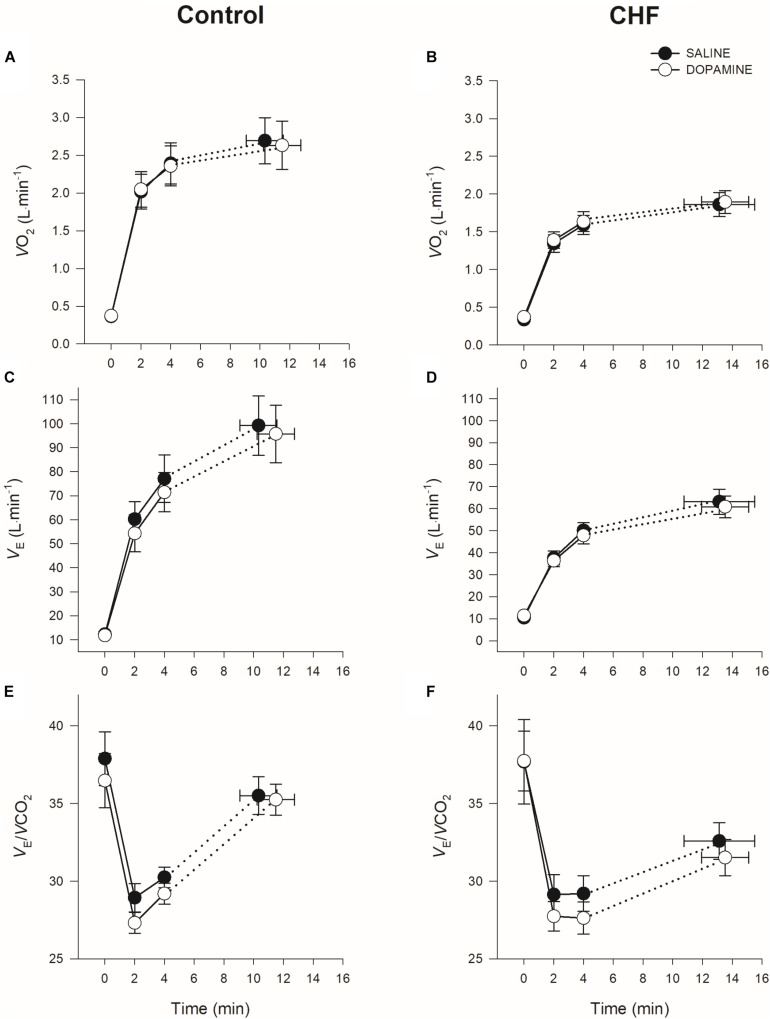
Mean ± SEM oxygen consumption (*V̇O*_2_), minute ventilation ($\dot{V}$_E_), and ventilatory efficiency ($\dot{V}$_E_/$\dot{V}$CO_2_) at rest and during constant-load cycle ergometry in controls (**A,C,E**) and CHF (**B,D,F**).

See Table 3, for the physiological and perceptual responses to dopamine during constant load exercise at 4-minute isotime, and Table 4, for the responses at TLIM. The effects of dopamine on *V̇O*_2_, $\dot{V}$_E_, and $\dot{V}$_E_*/V̇CO*_2_ during constant load exercise in controls and patients with CHF are displayed in Figure 1. $\dot{V}$O_2_, $\dot{V}$CO_2_, and $\dot{V}$_E_ were significantly lower in patients with CHF, independent of condition, when compared to controls (*p* < 0.001, *p* < 0.001, *p* < 0.001, respectively). There was no significant difference in *V̇O*_2_, *V̇CO*_2_, $\dot{V}$_E_ or $\dot{V}$_E_/*V̇CO*_2_ between conditions at isotime (2- and 4-minute) or TLIM in either group. Dopamine significantly increased P_ET_CO_2_ in CHF at baseline, 2- and 4-minute (*p* = 0.008, *p* = 0.007, *p* < 0.001, respectively), but not at TLIM (*p* = 0.26). In controls, dopamine significantly increased P_ET_CO_2_ at 2- and 4-minute (*p* = 0.032 and *p* = 0.022, respectively), but not at baseline or TLIM and (*p* = 0.31 and *p* = 0.56, respectively). Dyspnea was unaffected by dopamine throughout exercise in either group (main effect for condition *p* = 0.45).

### The Effects of Dopamine on the Cardiovascular Responses to Exercise

**FIGURE 2:**
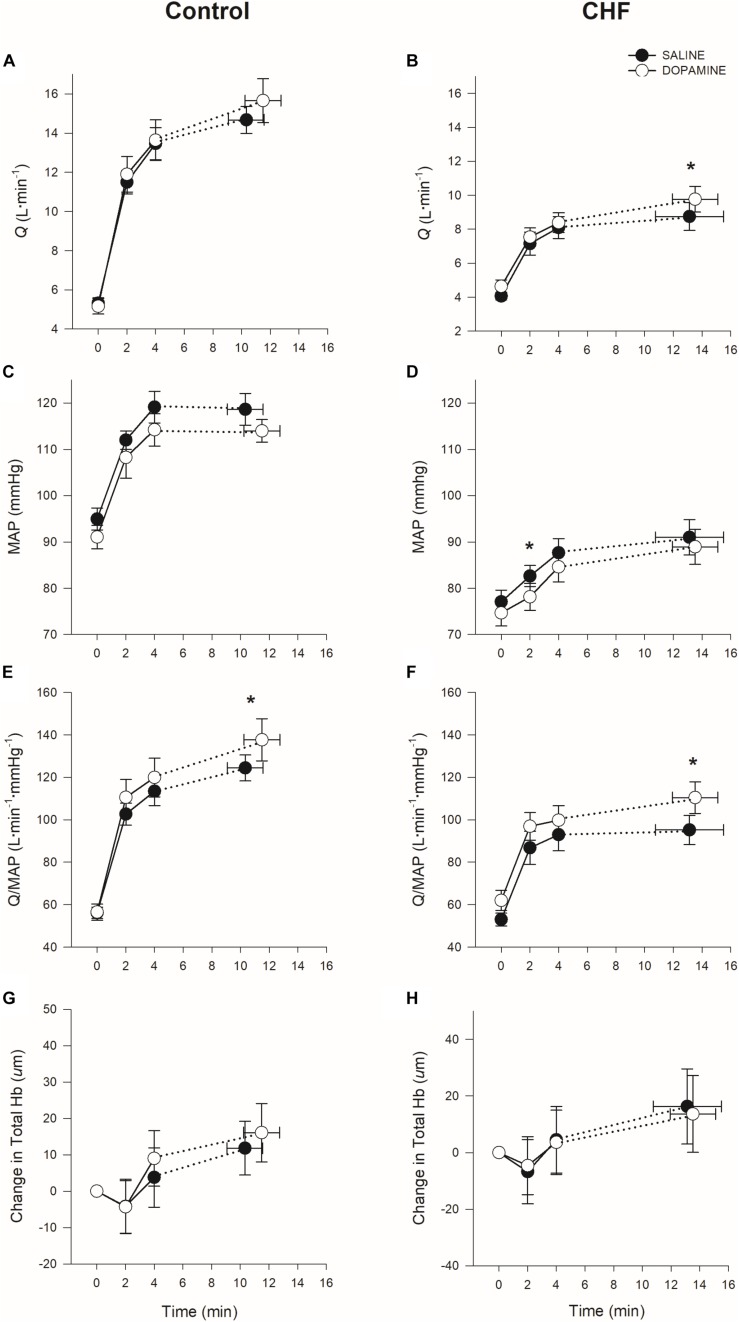
Mean ± SEM cardiac output (*Q*), mean arterial pressure (*MAP*), vascular conductance, and vastus lateralis tissue oxygentation [Total Hemoglobin (Hb)] at rest and during constant-load cycle ergometry in controls (**A,C,E,G**) and CHF (**B,D,F,H**). *p < 0.05 saline vs. dopamine within group.

The effects of dopamine on the cardiovascular responses to exercise can be found in Tables 3, (4-minute isotime) and 4 (TLIM), and Figure 2. Cardiac output, heart rate (HR), stroke volume (SV), MAP, and conductance were lower in patients with CHF than in controls independent of condition (Q: *p* < 0.001; HR: *p* < 0.001; SV: *p* < 0.001; MAP: *p* < 0.001; conductance: *p* < 0.001). Q was increased with dopamine in patients with CHF at TLIM (*p* = 0.03); likely secondary to a trend in increased SV (*p* = 0.05) while no change in HR was observed. Vascular conductance was significantly increased with dopamine in CHF (*p* = 0.01) as well as controls (*p* = 0.03) at TLIM, despite no significant changes in MAP in either group at TLIM (Figure 2). There were no significant changes in Q, SV or HR in controls with dopamine at 4-minute isotime or TLIM. At TLIM, DO_2_ was higher with dopamine in both groups (controls: *p* = 0.03 vs. CHF: *p* = 0.04). This improvement in DO_2_ at TLIM in controls was likely secondary to a trend in increased [Hb] with dopamine (*p* = 0.07), which was not observed in patients with CHF. Despite improved DO_2_ at peak exercise, tissue oxygenation at the vastus lateralis was unaffected by dopamine in either group (controls: *p* = 1.00 vs. CHF: *p* = 0.96). There were no changes in ratings of perceived leg discomfort in either group between both conditions.

## Discussion

To date, this is the first study to evaluate the effects of carotid chemoreceptor inhibition with low-dose dopamine on exercise tolerance, and cardiovascular and ventilatory regulation in patients with CHF; and our findings are twofold. First, CC inhibition with dopamine improved vascular conductance at TLIM in both groups. Further, dopamine increased Q and O_2_ delivery at TLIM in patients with CHF. Second, despite improvements in vascular conductance/O_2_ delivery, CC inhibition had no effect on exercise endurance time in either group. When combined, these findings suggest that the CC contributes to cardiovascular control during exercise in both health and CHF, but the CC does not contribute to exercise intolerance in CHF.

### The Effects of Dopamine on Cardiovascular Function During Exercise

It has been well documented that CC activity/sensitivity is increased in both experimental CHF (Sun et al., 1999a, b; Ponikowski et al., 2001; Stickland et al., 2007) and in patients with CHF (Chua et al., 1997; Sun et al., 1999b; Ponikowski et al., 2001; Stickland et al., 2007; Giannoni et al., 2008, 2009). Furthermore, it has been demonstrated that the CC contributes to the sympathetic restraint of exercising muscle blood flow both in health (Stickland et al., 2008, 2011) and experimental CHF (Stickland et al., 2007). Previous work has shown no CV effect of CC inhibition in CHF patients performing hand grip exercise (Edgell et al., 2015), however, the current study demonstrated that CC inhibition increased vascular conductance and peak Q during whole-body cycle exercise in CHF. Both central (reduced convective oxygen transport and Q) and peripheral factors may be involved in the exercise intolerance typically observed in CHF (Haykowsky et al., 2015). Peripheral factors limiting exercise in CHF may include: impaired local skeletal muscle blood flow (LeJemtel et al., 1986; Sullivan et al., 1989; Wilson et al., 1993; Esposito et al., 2010), impaired diffusive oxygen transport (Esposito et al., 2010, 2011) as well as skeletal myopathy (Coats et al., 1994). The improvement in Q with dopamine translated into an increase in oxygen delivery at TLIM in CHF. Despite the improvement in central convective oxygen transport with dopamine (i.e., increased DO_2_), there was no change in exercise endurance time in patients with CHF. Both vastus lateralis THb (index of local blood flow) and HHb (index of O_2_ extraction) were unaffected by dopamine in patients with CHF. It is possible that the sympathetic mediated improvement in total vascular conductance (i.e., vasodilation) and global O_2_ delivery with dopamine did not translate to improved leg blood flow and oxygen delivery. Additionally, the lack of effect of CC inhibition on vastus lateralis HHb, despite improved central oxygen transport during exercise supports previous evidence that patients with CHF have a peripheral limitation to exercise (LeJemtel et al., 1986; Sullivan et al., 1989; Wilson et al., 1993; Coats et al., 1994; Esposito et al., 2010, 2011; Bhella et al., 2011). Because THb and HHb were both unaffected with dopamine in patients with CHF, it is difficult to partition out whether a sympathetic–mediated restraint in blood flow or an impairment in vastus lateralis O_2_ extraction prevented an improvement in tissue oxygenation and ultimately exercise tolerance (despite improved central oxygen transport). Future experiments directly measuring leg blood flow and conductance are required to better understand the complex relationship between sympathetic mediated restraint of locomotor blood flow, muscle O_2_ diffusion and extraction in CHF.

### The Effects of Dopamine on Ventilation During Exercise

It has been well established that patients with CHF have an exaggerated ventilatory response to exercise (Weber et al., 1982; Sullivan et al., 1988; Myers et al., 1992; Koike et al., 1993; Riley et al., 1994; Kobayashi et al., 1996), which can contribute to the sensation of dyspnea (Rubin and Brown, 1984). The elevated CC sensitivity typically observed in CHF has been linked to the heightened ventilatory response to exercise (Chua et al., 1996a). Despite evidence of enhanced carotid chemosensitivity, the CHF patients in the current study did not demonstrate an exaggerated $\dot{V}$_E_/$\dot{V}$CO_2_ response to exercise, and CC inhibition with dopamine did not reduce minute ventilation or dyspnea during exercise in CHF. We would suggest that the absence of an exaggerated $\dot{V}$_E_/$\dot{V}$CO_2_ in patients with CHF during exercise could be due to the pharmacological management of these patients; β-blockers have been shown to lower $\dot{V}$_E_/$\dot{V}$CO_2_ in CHF but do not affect the CC (Beloka et al., 2008). We did observe an increase in P_ET_CO_2_ at baseline and isotime in CHF patients with CC inhibition, while in controls, P_ET_CO_2_ was unaffected by dopamine at baseline, but increased at isotime. The observed increase in P_ET_CO_2_, would suggest a relative alveolar hypoventilation, secondary to CC inhibition. These data suggest that although the CC may help with matching alveolar ventilation to metabolic demand, the heightened basal CC sensitivity does appear to contribute to elevated minute ventilation, dyspnea and exercise intolerance in CHF.

### Methodological Considerations

Historically, work in experimental CHF (Sun et al., 1999b; Li et al., 2006, 2007; Stickland et al., 2007; Ding et al., 2008; Marcus et al., 2014) has been on animals that have pacing-induced (i.e., chronic ischemia) CHF, and these animals typically do not receive CV medications to help manage their disease, nor do they typically have co-morbidities. As a result, there are significant limitations related to the translation of previous findings in experimental models of CHF to patients with CHF. Additionally, there is variability in humans with CHF in terms of: comorbidities, HF etiology, and emerging pharmacotherapies being used in patients with CHF. In the current study, both patients with HFpEF and HFrEF were examined. While there were no apparent differences with CC inhibition between the two etiologies, this would need to be confirmed with a properly designed comparison study.

Original work demonstrating enhanced CC activity/sensitivity in CHF was completed on patients with lower EF (HFREF) and higher NYHA functional class (Chua et al., 1996a, b, 1997; Ponikowski et al., 2001) than the patients in the current study. Further, patients in these previous studies were not treated with β-blockers or ARBs but were receiving digoxin. Digoxin is no longer the preferred treatment for patients with CHF (Lewis et al., 1989), and has been shown to sensitize the CC (Quest and Gillis, 1971; McQueen and Ribeiro, 1983; Schobel et al., 1994; Janssen et al., 2010). Future clinical trials investigating the effect of CC-mediated CV function before and after treatment may provide further insight into the benefits of pharmacotherapy on CC function.

To our knowledge, there is no minimally clinically important difference (MCID) in exercise endurance time with constant work-rate exercise in CHF, but the MCID in COPD has been determined to be 101 s (Puente-Maestu et al., 2009). The current study found a 23.5 ± 83.2 s improvement in exercise endurance time with dopamine in patients with CHF, which suggests that the current observed effect size is unlikely to be of physiological or clinical significance in CHF. Further, a *post hoc* sample size calculation was completed based on the current mean difference in exercise endurance time (mean ± SD: 0.39 ± 4.8 min), and estimated that 1184 patients with CHF would be required to detect a significant effect of CC inhibition with dopamine in exercise tolerance (effect size = 0.08, α = 0.05, β = 0.2, power = 0.8). Based on the small absolute increase with dopamine and the *post hoc* power calculation, we would suggest that the inability to detect a difference in exercise endurance time with dopamine is unlikely the result of being statistically underpowered.

Limberg et al. (2016) found that there was individual variability as to the most effective dose of dopamine to inhibit the CC. In the current study, we used a standardized dose of IV dopamine hydrochloride (2 μg⋅kg^–1^⋅min^–1^) that has been previously used by our group and shown to inhibit the CC without resulting in alpha- or beta-adrenergic stimulation (Stickland et al., 2011; Edgell et al., 2015; Phillips et al., 2018). It is possible that by using a dose-response curve, we may have found a more effective individual dose of dopamine for each patient. However, being concerned about alpha-adrenergic stimulation with higher doses of dopamine (which could increase SNA and vasoconstriction) (Hoffman and Lefkowitz, 1990; Ciarka et al., 2007), we decided to use a conservative dose of dopamine hydrochloride (2 μg⋅kg^–1^⋅min^–1^).

It is generally assumed that low-dose dopamine directly stimulates dopamine-1 vascular receptors eliciting a vasodilatory response (Goldberg et al., 1963; Davis et al., 1982; Varriale and Mossavi, 1997; Elkayam et al., 2008). Work to date has shown that low-dose dopamine causes vasodilation in conditions of high CC activity/sensitivity such as CHF (Stickland et al., 2007; Edgell et al., 2015) and COPD (Phillips et al., 2018). To our knowledge, vasodilation does not occur with low-dose dopamine infusion in healthy participants where CC activity/sensitivity is normal (Lahiri et al., 1980; Stickland et al., 2011; Phillips et al., 2018). While is it possible that the vasodilation observed with dopamine could be due to the direct peripheral vascular actions of dopamine; any peripheral vascular effects of dopamine would have been consistently observed throughout exercise in both groups. Importantly, direct stimulation of dopamine-1 vascular receptors would not affect ventilatory control. We have previously shown that the identical dose of dopamine used in the current study inhibits resting minute ventilation in CHF patients, but not healthy controls (Edgell et al., 2015). In the current study, dopamine increased P_ET_CO_2_ (indicating a small reduction in alveolar ventilation) at baseline immediately prior to exercise in CHF patients, and during exercise in both controls and CHF. When combined, these results suggest that the cardiovascular effects of dopamine observed in the current study are secondary to CC inhibition and not a direct peripheral vascular effect.

## Conclusion

In conclusion, this study examined the effect of CC inhibition using low-dose dopamine on exercise tolerance, and cardiovascular and ventilatory function in patients with CHF and healthy controls. CC inhibition improved vascular conductance during exercise in both groups and increased peak cardiac output and oxygen delivery in CHF, but this did not translate to improved exercise tolerance in either group. Additionally, dopamine did not affect minute ventilation nor dyspnea during exercise in patients with CHF, or controls. Importantly, this work suggests that the CC contributes to CV regulation in both patients with optimally treated chronic heart failure and healthy controls during whole-body exercise.

## Data Availability Statement

The datasets generated for this study are available on request to the corresponding author.

## Ethics Statement

The studies involving human participants were reviewed and approved by University of Alberta Health Research Ethics Board - Biomedical Panel study ID No. Pro00000526. The patients/participants provided their written informed consent to participate in this study.

## Author Contributions

SC, DP, MF, and MS contributed to the conception or design of the work SC, DP, MM, TB, DP, JE, EW, and MS contributed to the acquisition, or analysis or interpretation of data for the work. All authors drafting the work or revising it critically for important intellectual content, approved the final version of the manuscript, and agree to be accountable for all aspects of the work. All persons designated as authors qualify for authorship, and all those who qualify for authorship are listed. This research was conducted through the Clinical Physiology Laboratory, Alberta Cardiovascular and Stroke Research Centre (ABACUS), Mazankowski Alberta Heart Institute.

## Disclaimer

The views expressed in the submitted article are of our own and not an official position of the institution.

## Conflict of Interest

The authors declare that the research was conducted in the absence of any commercial or financial relationships that could be construed as a potential conflict of interest.
